# *Mycobacterium tuberculosis* whole genome sequencing provides insights into the Manila strain and drug-resistance mutations in the Philippines

**DOI:** 10.1038/s41598-019-45566-5

**Published:** 2019-06-26

**Authors:** Jody E. Phelan, Dodge R. Lim, Satoshi Mitarai, Paola Florez de Sessions, Ma. Angelica A. Tujan, Lorenzo T. Reyes, Inez Andrea P. Medado, Alma G. Palparan, Ahmad Nazri Mohamed Naim, Song Jie, Edelwisa Segubre-Mercado, Beatriz Simoes, Susana Campino, Julius C. Hafalla, Yoshiro Murase, Yuta Morishige, Martin L. Hibberd, Seiya Kato, Ma. Cecilia G. Ama, Taane G. Clark

**Affiliations:** 1 Infection Biology Department, London School of Hygiene and Tropical Medicine, London, WC1E 7HT UK; 2 National Tuberculosis Reference Laboratory, Research Institute for Tropical Medicine, Muntinlupa City, Philippines; 3 TB Study Group, Research Institute for Tropical Medicine, Muntinlupa City, Philippines; 4 Department of Mycobacterium Reference and Research, Research Institute of Tuberculosis, Japan Anti-Tuberculosis Association, Tokyo, Japan; 5 Genome Institute of Singapore, 60 Biopolis St, Biopolis, Singapore; 6 Molecular Biology Laboratory, Research Institute for Tropical Medicine, Muntinlupa City, Philippines; 7 Department of Infectious Disease Epidemiology, Faculty of Epidemiology and Population Health, London School of Hygiene and Tropical Medicine, London, WC1E 7HT UK

**Keywords:** Bacterial genomics, Antimicrobial resistance

## Abstract

The Philippines has a high incidence of tuberculosis disease (TB), with an increasing prevalence of multidrug-resistant *Mycobacterium tuberculosis* (MDR-TB) strains making its control difficult. Although the *M*. *tuberculosis* “Manila” ancient lineage 1 strain-type is thought to be prevalent in the country, with evidence of export to others, little is known about the genetic diversity of circulating strains. By whole genome sequencing (WGS) 178 isolates from the Philippines National Drug Resistance Survey, we found the majority (143/178; 80.3%) belonged to the lineage 1 Manila clade, with the minority belonging to lineages 4 (European-American; n = 33) and 2 (East Asian; n = 2). A high proportion were found to be multidrug-resistant (34/178; 19.1%), established through highly concordant laboratory drug susceptibility testing and *in silico* prediction methods. Some MDR-TB isolates had near identical genomic variation, providing potential evidence of transmission. By placing the Philippine isolates within a phylogeny of global *M*. *tuberculosis* (n > 17,000), we established that they are genetically similar to those observed outside the country, including a clade of Manila-like strain-types in Thailand. An analysis of the phylogeny revealed a set of ~200 SNPs that are specific for the Manila strain-type, and a subset can be used within a molecular barcode. Sixty-eight mutations known to be associated with 10 anti-TB drug resistance were identified in the Philippine strains, and all have been observed in other populations. Whilst nine putative streptomycin resistance conferring markers in *gid* (8) and *rrs* (1) genes appear to be novel and with functional consequences. Overall, this study provides an important baseline characterisation of *M*. *tuberculosis* genetic diversity for the Philippines, and will fill a gap in global datasets and aid the development of a nation-wide database for epidemiological studies and clinical decision making. Further, by establishing a molecular barcode for detecting Manila strains it will assist with the design of diagnostic tools for disease control activities.

## Introduction

Tuberculosis disease (TB) is a chronic respiratory infection, caused by *Mycobacterium tuberculosis*. The Philippines is a high-burden country for TB, with over 300,000 cases and 25,000 deaths in 2017 alone^1^. However, recent findings suggest that over a million Filipinos are actively infected with the disease, placing the Philippines burden second to China^1^. Furthermore, the country has an increasing HIV prevalence and a high burden for multidrug-resistance (MDR-TB, resistance to isoniazid and rifampicin (RIF) treatments) that pose serious challenges for effective control^1^. The Philippines has adopted the World Health Organisation (WHO) TB - “Directly Observed Treatment, Short Course” (TB-DOTS) control strategy. It has been recognised that there is an urgent need to detect and treat more cases through an expansion of GeneXpert (Cepheid, Sunnyvale, CA) facilities for better access and for use as a primary diagnostic tool^1^. In particular, there is a gap among those expected to have MDR-TB (2% new, 21% retreatment cases) and those detected and subsequently put on treatment. Furthermore, treatment outcomes among those treated show poor success rates, thereby putting the country at risk to the further spread of MDR-TB and potentially extensively drug-resistant strains.

Despite TB being a serious problem in the Philippines, genomic data for local *M*. *tuberculosis* strains is lacking. Previous studies have identified ‘ancient’ (lineage 1) and ‘modern’ lineages (2 and 4) as being present in the population^2^ with the members of the EA12-Manila clade known to be highly associated with the Filipino population^3,4^. More isolates are needed to accurately infer the phylogeographic distribution of *M*. *tuberculosis* strains in the country, and take advantage of new genomic tools that allow for whole genome sequencing (WGS). WGS data can be used to profile the *M*. *tuberculosis* for drug resistance^5,6^, characterise the ancient and modern lineages and different virulence strain-types^7^, and establish who may have transmitted to whom and thus allow targeted resources to hotspot areas to reduce transmission^8^; all made possible through advances in health informatics^5^. Further, WGS opportunities are set to revolutionise the diagnosis and clinical patient management of TB. For example, Public Health England (UK) now uses pathogen genetic characterisation as a clinical standard of care in TB management, and an increasing number of countries worldwide are seeking to adopt this as part of clinical care. However, to be effective these approaches need to be applied in countries with high TB burdens and coordinated with established control programmes.

In the Philippines, a nationwide drug resistance survey (DRS) occurs quinquennially (years 2007, 2012, 2017) and there is active surveillance of MDR-TB, including monitoring of fluoroquinolone and second line injectables resistance among TB patients, which involves  the sequencing of drug resistance loci. This involves characterising single nucleotide polymorphisms (SNPs) and indel markers of drug resistance, especially in genes coding for drug-targets or -converting enzymes (e.g. *katG*, *inhA*, *rpoB*, *pncA*, *embB*, *rrs*, *gyrA*, *gyrB* genes). Limited sets of high frequency markers have been included in diagnostic tools (e.g. Xpert MTB/RIF and line probe assays for MDR-TB) for identification in *M*. *tuberculosis* DNA (approximate genome size 4.4 Mb). However, these approaches use less than 1% of the genome, and WGS approaches that identify SNPs and other variation provide far greater resolution for profiling *M*. *tuberculosis* for drug resistance, strain-types and detecting outbreaks, but also inform new mutations and mechanisms through genome-wide analyses^6,9^.

To date, there have been few WGS studies in the Philippines, and here we present the results of sequencing 178 isolates recently collected in the 2012 DRS. We explore the genomic diversity of predominant Manila strain and develop a molecular barcode for surveillance applications. We show that the strain-types and majority of drug resistance mutations identified are similar to those found in global populations, making the use of existing mutation databases robust to detect resistance.

## Results and Discussion

### Clinical isolates and phylogeny

**Figure 1:**
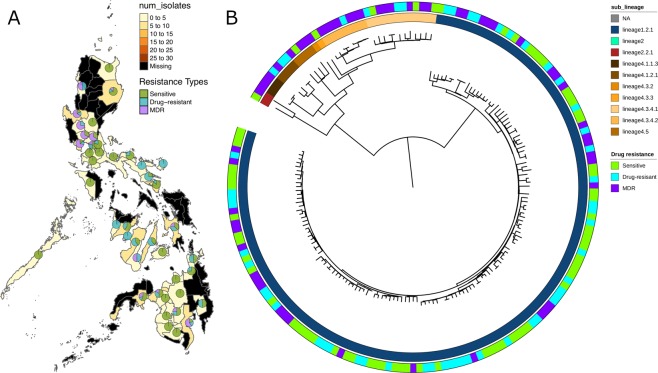
The 178 *M*. *tuberculosis* isolates: (**A**) Map of the Philippines annotated with the source and drug-resistance of the isolates; (**B**) A phylogenetic tree constructed using 17,522 SNPs.

A total of 178 *M*. *tuberculosis* isolates were sourced from the nationwide “cluster-based”, cross-sectionally designed TB DRS (2012), which was implemented with cross re-checking standards according to the WHO guidelines. The isolates were randomly selected to represent the different geographic regions (“clusters”) in the Philippines. (Fig. 1A; Table 1). The isolates underwent culture, drug susceptibility testing (DST) across 8 drugs (Table 1), and WGS. The DST results revealed 97 (54.5%) isolates as pan-susceptible, 34 (19.1%) MDR-TB (resistance to both rifampicin and isoniazid) and 47 (26.4%) non-MDR-TB drug-resistant (resistance to at least one drug but not to both rifampicin and isoniazid). No XDR-TB isolates were found, confirming earlier reports of low levels of fluoroquinolone resistance^10^. Mapping of the raw sequence data led to high average genome-wide coverage across the samples (median: 100.5-fold; range: 52- to 2,263-fold). Across the clinical isolates, 17,522 unique SNPs were identified, and a high proportion (50%) were observed in single isolates. Isolates were classified into lineages 1 (n = 143; 80.3%), 4 (n = 33; 18.5%) and 2 (n = 2; 1.1%), and as expected these form clusters on the phylogeny constructed using all SNP sites (Fig. 1B). By inferring spoligotypes from the WGS data, the EA12-Manila spoligotype (lineage 1.2.1)^3^ was found to have the highest frequency (n = 143, 80.3%) among lineage 1 strains (Table 1).

**Table 1:** *Mycobacterium tuberculosis* samples (N = 178).

Characteristic	N	%
Location*		
Luzon	105	59.0
Visayas	37	20.8
Mindanao	36	20.2
*Lineages*		
*1*	143	**80.3**
*2*	2	**1.1**
*4*	33	**18.5**
Drug resistance**		
Rifampicin	38	**24.7**
Isoniazid	64	**46.0**
Ethambutol	10	**6.5**
Streptomycin	25	**16.3**
Amikacin	2	**1.1**
Capreomycin	2	**1.2**
Kanamycin	8	**4.5**
Pan-susceptible	97	**54.5**
MDR-TB	34	**21.5**
XDR-TB	0	**0**

*Island of collection for sequenced isolates.

**The number resistant to the drug and percentage of tested isolates resistant; the denominator is the number of isolates for each drug with successfully performed susceptibility tests. The exception to this is the proportion of pan-susceptible which was calculated using the total number of samples.

MDR-TB = multi-drug resistant; XDR-TB = extensively-drug resistant; there was no levofloxacin drug resistance.

### Evidence of transmission

Potential transmission clusters were found by calculating the pairwise SNP distance between isolates and using an established difference cut-off of 10 SNPs or less^8^. Fifteen clusters were found, with a  maximum size of three isolates. Of these clusters, nine contained MDR-TB strains, ten belonged to lineage 1, and five to lineage 4 (Supplementary Fig. 2). In order to establish whether particular strains are circulating locally or nation-wide, the phylogeographical distribution of the 178 strains was analysed (Supplementary Fig. 3A). There was no association of island group (Luzon, Mindanao and Visayas) with phylogenetic placement (Slatkin-Maddison P = 0.87). Using the closest neighbour for each strain, a weak correlation between genetic distance (SNP differences) and geographic distance was observed (Spearmans correlation = 0.36) (Supplementary Fig. 3B).

### The Manila strain

This collection represents the largest number of Manila strains sequenced from the Philippines with WGS (n = 143). To determine if the Philippine strains formed their own monophyletic clade compared to other strains, we reconstructed a phylogenetic tree that also included lineage 1.2.1 strains with publicly available sequences (n = 408). The Philippine isolates did not form a monophyletic group within the tree and were interleaved with strains from Thailand, UK, Saudi Arabia, India, and Vietnam among others (Supplementary Fig. 4). Most of these were sporadically scattered throughout the tree, however a large monophyletic clade with 114 isolates formed comprising of predominantly Thai strains. To determine the geographic origin of the most recent common ancestor (MRCA) of this clade, we performed ancestral state reconstruction using stochastic character mapping. The most likely location of the Thai clade MRCA was estimated to be from the Philippines (posterior probability = 0.79). Based on this, a likely scenario is that lineage 1.2.1 strains in Thailand were imported from the Philippines, after which they diversified. *In-silico* spoligotyping of this clade revealed a very different strain-type from the Manila spoligotype, with the loss of spacers 7–18 and 21–24, corresponding to the previously described EAI2-Nonthaburi spoligotype^11^. A comparison of allele frequency differences between the Manila (N = 408) and non-Manila strains in neighbouring clades (N = 1,636) revealed 197 SNPs which were specific to the Manila clade. Of these, 180 were found in coding regions and 115 led to amino acid changes, including one premature stop codon in *whiB5*, which has been described before^12^ (Supplementary Data 1).

### Mutations underlying drug-resistance

Resistance profiles were predicted *in silico* using an established library of mutations (https://github.com/jodyphelan/tbdb), and were compared to DST results. Assuming DST as the gold standard, the sensitivity and specificity was calculated for rifampicin, isoniazid, ethambutol and streptomycin, where all had at least 10 isolates with demonstrated phenotypic resistance. The sensitivity of prediction was high (>90%) across the drugs except streptomycin. The specificity was high (>95%) for all drugs (Supplementary Table 1). The relative low sensitivity of the streptomycin predictions was investigated and revealed nine novel polymorphisms in the *gid* (8 mutations) and *rrs* (1 mutation) genes in 12 false negative isolates, which could potentially confer drug resistance. To investigate these mutations, we looked at their frequency in a large dataset of ~17,000 publicly available strains. Of the nine mutations, six were found in the global dataset and only one of these was found to be a lineage-specific marker (*gid* L16R) with the other five displaying characteristics of convergent evolution. The functional consequences of the mutations in *gid* were investigated, and it was established that two (W45*, S181*) led to premature stop codons, two (CA49C, AC194A) led to frameshifts, and three (G71R, V77G and D85G) were within or close to the S-adenosyl-L-methionine (SAM)-binding motif. The SAM-binding motif is an important functional domain required by *gid* to methylate the G527 on the 530 loop of 16S rRNA, which is thought to be the binding location of streptomycin^13^ (Supplementary Fig. 5). These mutations could represent resistance conferring mutations and should be investigated further. Overall, 68 known drug resistance-related mutations were found across the 178 samples, and they varied in frequency (range: 1 to 63 isolates) (Table 2). Although no phenotypic resistance to fluoroquinolones was found, one isolate had the N499K mutation in the *gyrB* gene. This mutation has been reported to be rare in clinical strains^14^ and is associated with resistance to moxifloxacin but not to ofloxacin or levofloxacin^15^. Predicted resistance to (non-streptomycin) aminoglycosides was also rare with only a single isolate presenting a resistance conferring mutation in *rrs* (A1401G).

**Table 2:** Drug resistance mutations with frequency greater than one.

Drug	Gene	Mutation	Freq. (%)	Global freq* (%)
Isoniazid	*katG*	S315T	63	4057/5423 (74.8)
Rifampicin	*rpoB*	S450L	37	2968/4618 (64.3)
Isoniazid/Ethionamide	*inhA promoter*	C-15T	35	872/5423 (16.1)
Ethambutol	*embB*	M306I	15	564/2662 (21.2)
Streptomycin	*rrs*	A514C	11	128/1408 (9.1)
Streptomycin	*rpsL*	K88R	7	103/1408 (7.3)
Rifampicin	*rpoB*	H445Y	6	230/4618 (5.0)
Isoniazid	*inhA*	I21V	5	15/5423 (0.3)
Ethambutol	*embB*	M306V	4	839/2662 (31.5)
Isoniazid/Ethionamide	*inhA promoter*	T-8C	4	89/5423 (1.6)
Ethambutol	*embB*	Q497K	3	20/2662 (0.8)
Isoniazid/Ethionamide	*inhA promoter*	G-17T	3	72/5423 (1.3)
Isoniazid/Ethionamide	*inhA*	I21T	3	32/5423 (0.6)
Streptomycin	*gid*	102del	3	12/1408 (0.9)
Streptomycin	*gid*	351del	3	6/1408 (0.4)
Streptomycin	*rpsL*	K43R	3	642/1408 (45.6)
Ethambutol	*embA*	D4N	2	3/2662 (0.1)
Ethambutol	*embB*	D328G	2	16/2662 (0.6)
Ethambutol	*embB*	G406D	2	75/2662 (2.8)
Isoniazid/Ethionamide	*inhA*	I194T	2	88/5423 (1.6)
Pyrazinamide	*pncA*	D12A	2	11/1946 (0.6)
Pyrazinamide	*pncA*	G24D	2	3/1946 (0.2)
Pyrazinamide	*pncA*	I133T	2	24/1946 (1.2)
Rifampicin	*rpoB*	1297_1298insTTC	2	11/4618 (0.2)
Rifampicin	*rpoB*	1295_1303del	2	5/4618 (0.1)
Rifampicin	*rpoB*	D435F	2	23/4618 (0.5)
Rifampicin	*rpoB*	S441L	2	17/4618 (0.4)
Streptomycin	*gid*	386del	2	2/1408 (0.1)
Streptomycin	*rrs*	C517T	2	57/1408 (4.1)

^*^Based on the 17k dataset.

### Summary

WGS is being used as a tool for epidemiological investigations and to assist TB clinical and control program decision making. However, most clinical applications of WGS are taking place in developed countries. We present the first WGS data from the nation-wide TB drug resistance survey in the Philippines. Most isolates were from the EAI2-Manila clade, which would make this the largest WGS study of this spoligotype, but is also indicative that this clade is common. For comparison, a recent report of WGS of 10 Philippines samples included 7 EAI2-Manila, 1 Beijing and 2 Euro-American strains^2^. In our WGS data, there was high SNP variability between isolates in the EAI2-Manila clade, and we identified 197 SNPs specific to the EAI2-Manila clade which could form the basis of a SNP barcode. There was some evidence of near identical isolates, which is indicative of transmission events. This observation is surprising as the samples were chosen pseudo-randomly from a large collection, but it may be indicative of many large transmission chains in the population. Future large-scale sequencing of isolates across the surveys is required to understand transmission patterns and dynamics. There is no evidence of a correlation between genetic and geographic distance, but this may reflect the within-country movement and non-localised transmission of strains in the Philippines. Finally, we performed *in-silico* drug resistance profiling and compared this to phenotypic DST results. We found a number of different mutations conferring resistance to first-line anti-TB drugs, and report novel potential resistance conferring mutations, which can be investigated experimentally. Further, the mutations observed are similar to those reported in other drug resistant *M*. *tuberculosis* from the Philippines^2^ and globally. Overall, our work reveals the utility of WGS for the prediction of drug resistance in the Philippines setting. The whole genome data generated will serve as a reference for the surveillance of TB in the Philippines and the wider region.

## Materials and Methods

### DNA extraction and sequencing

A total of 178 *M*. *tuberculosis* short-term cultured isolates from sputum samples collected from the second national DRS on TB in the Philippines (2012) were pseudo-randomly selected for the study, where selection was informed by the WHO cross re-checking standard. This study was given authorization by the Institutional Review Board of the Research Institute for Tropical Medicine in the Philippines (ID No. RITM-IRB 2017-05). The clinical samples were complemented by two replicates of the H37Rv reference strain. Drug susceptibility testing was performed for rifampicin, isoniazid, ethambutol, streptomycin, amikacin, kanamycin, capreomycin and levofloxacin at the  National Tuberculosis Research Laboratory (NTRL) (Supplementary Table 1). For the H37Rv and 14 clinical isolates, inactivation of culture isolates was performed in a BSL-3 facility at the NTRL,  and DNA extraction at the Molecular Biology Laboratory at the Research Institute for Tropical Medicine (RITM). Total genomic DNA was extracted using a phenol-chloroform extraction procedure. DNA pellets were washed with 70% ethanol and resuspended in 100ul Buffer EB (QIAGEN Inc., Germantown, MD, USA). DNA extract concentration and quality were measured using a Qubit fluorometer (Life Technologies Holdings Pte Ltd, Singapore) and were visualized on a 1% agarose gel. Library preparation of the DNA samples was performed using QIAseq FX DNA library kit, following the manufacturer’s protocol. Agilent High Sensitivity DNA Kit (QIAGEN Inc., Germantown, MD, USA) was used to check for correct DNA fragment size after adapter ligation. The concentration and size of each DNA library was measured using Agilent Technologies DNA 1000 assay kit on Agilent Technologies 2100 Bioanalyzer. The H37Rv prepared DNA libraries were sequenced using Illumina HiSeq. 4000 Next Generation Sequencer (2 × 151 base pair reads) at the Genome Institute Singapore. Similarly, for the other 164 clinical strains, DNA extraction and purification were performed in the BSL-3 laboratory in the Research Institute of Tuberculosis (RIT) using Isoplant II (WAKO chemicals, Japan). The clinical strains were sequenced on the Illumina MiSeq platform (350 forward and 250 reverse pair ends) at the RIT. All raw sequencing data is available (Supplementary Data 2).

### Bioinformatic analyses

Sequence reads were inspected using fastQC (www.bioinformatics.babraham.ac.uk/projects/fastqc/) as a primary assessment of data quality. The reads were trimmed using trimmomatic^16^ (v0.38) to remove low quality sequences, and then mapped against the H37Rv reference genome (AL123456) using BWA (v0.7.17)^17^. SNPs were called using the BCF/VCF tool suite (v1.8)^18^ in regions where at least 10 reads were present. SNPs were removed from non-unique regions of the genome (e.g. *ppe* genes). SNPs were converted into a FASTA format alignment, which was used as input to RAxML (v8.0.0)^19^ to reconstruct the phylogeny. Drug resistance profiles and lineages were predicted *in-silico* using TBProfiler (v2.0). SpolPred (v1)^20^ was used to *in-silico* predict spoligotypes. As expected the resolution from using spoligotypes alone did not have the power to differentiate distinct WGS-based clusters found on the phylogenetic tree (Supplementary Fig. 1). The Philippine isolates were compared to others in a global set^6^ with the same lineage (see Supplementary Data 3) and F_ST_ values were calculated for each SNP using vcftools (–weir-fst-pop) to identify Manila-strain specific markers. A Slatkin-Maddison test was applied to assess a geographic-phylogenetic association and was performed in R using the prmac/slatkin.maddison library.

## Supplementary information

Supplementary materials

Supplementary data 2

Supplementary data 3

Supplementary data 1

## Data Availability

Previously published and newly generated data can be found on the ENA using the Run accession codes in Supplementary Data 2. The newly generated data can be found under the ENA study accession number ERP110368.
