# Supplementary material for: Mycobacterium tuberculosis whole genome sequencing provides insights into the Manila strain and drug-resistance mutations in the Philippines
Source: Sci Rep. 2019 Jun 26;9:9305. doi: 10.1038/s41598-019-45566-5 (PMC6594935; doi:10.1038/s41598-019-45566-5)
Supplement: Supplementary file 1 — Supplementary materials [file 41598_2019_45566_MOESM1_ESM.pdf]

# *Mycobacterium tuberculosis* whole genome sequencing provides insights into the Manila strain and drug-resistance mutations in the Philippines

Jody E. Phelan[1,*], Dodge R. Lim[2,3,*], Satoshi Mitarai [4],Paola Florez de Sessions[5], Ma. Angelica A. Tujan[6], Lorenzo T. Reyes[2,3], Inez Andrea P. Medado[6], Alma G. Palparan[2,3], Ahmad Nazri Mohamed Naim[5], Song Jie[5], Edelwisa Segubre-Mercado[6], Beatriz Simoes[1], Susana Campino[1], Julius C. Hafalla [1], Yoshiro Murase[4], Yuta Morishige[4], Martin L. Hibberd [1], Seiya Kato[4], Ma. Cecilia G. Ama[2,3,**], Taane G. Clark[1,7,**]

1 Infection Biology Department, London School of Hygiene and Tropical Medicine, London WC1E 7HT, UK

2 National Tuberculosis Reference Laboratory, Research Institute for Tropical Medicine, Muntinlupa City, Philippines

3 TB Study Group, Research Institute for Tropical Medicine, Muntinlupa City, Philippines

4 Department of Mycobacterium Reference and Research, Research Institute of Tuberculosis, Japan Anti-Tuberculosis Association, Japan

5 Genome Institute of Singapore, 60 Biopolis St, Singapore

6 Molecular Biology Laboratory, Research Institute for Tropical Medicine, Muntinlupa City, Philippines

7 Department of Infectious Disease Epidemiology, Faculty of Epidemiology and Population Health, London School of Hygiene and Tropical Medicine, London WC1E 7HT, UK

* Joint authors

** Joint authors

Corresponding author:

Professor Taane G. Clark

Pathogen Molecular Biology Department

Faculty of Infectious and Tropical Diseases

London School of Hygiene and Tropical Medicine, London WC1E 7HT, UK

Taane.clark@lshtm.ac.uk

**Supplementary figure 1**

**Resolution differences between clusters found using whole genome sequencing (WGS) and spoligotyping data.**

**A) Clusters found through phylogenetic reconstruction using SNPs derived from WGS data; B) clusters found through minimum spanning tree reconstructed from spoligotype haplotypes.**

**Potential subclades are labelled using the same colours.**

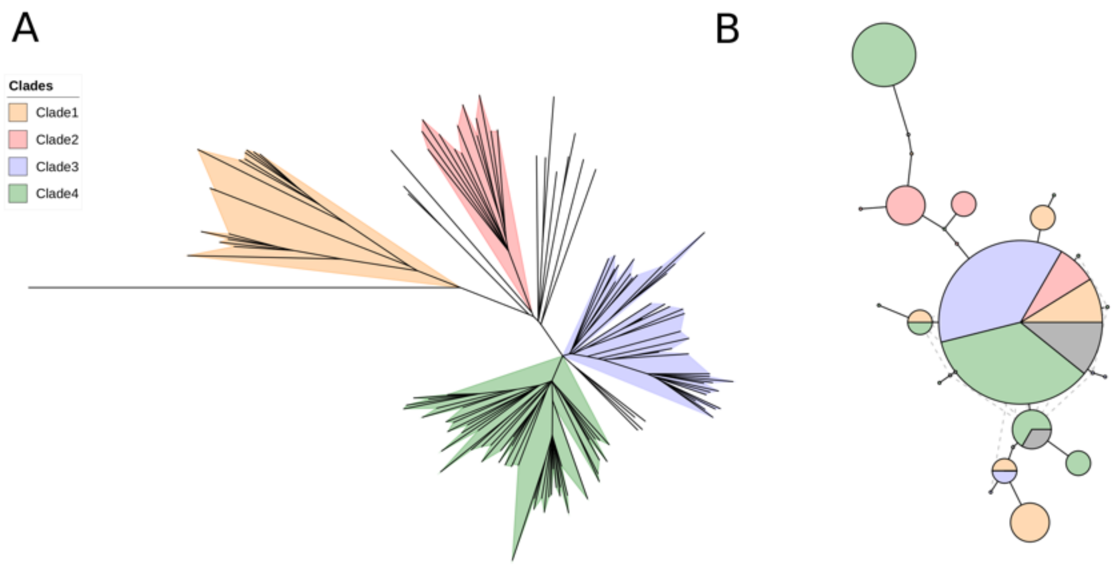

**Supplementary figure 2**

**Potential transmission clusters observed using whole genome sequencing data, where linked samples have 10 or less SNPs difference**

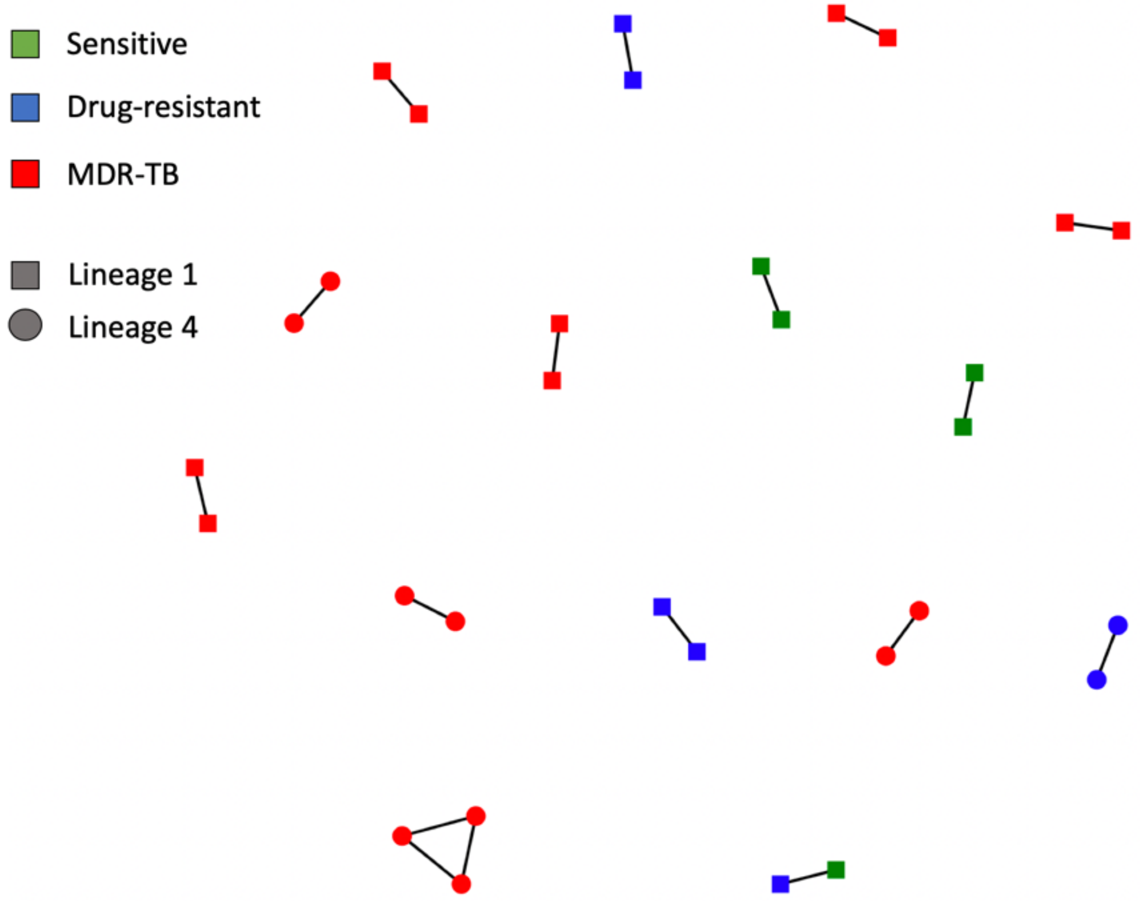


MDR-TB = multi-drug resistant

**Supplementary figure 3**

***M. tuberculosis* geography and phylogeny: A) A phylogenetic tree with the leaves linked to collection points; B) The relationship between genetic and geographical distances, with a (red) smoothed spline fit (Spearman's correlation 0.36)**

**A)**

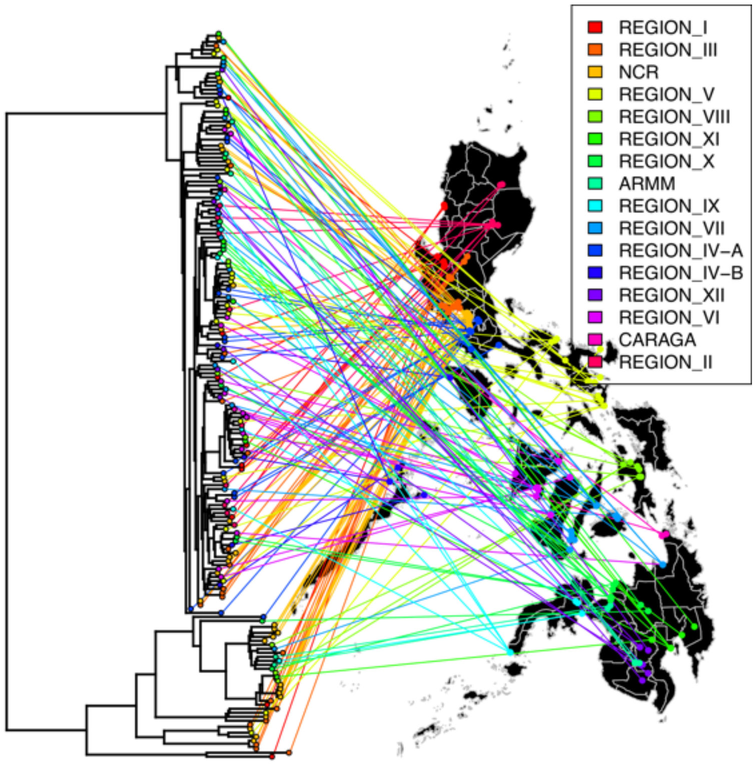


**B)**

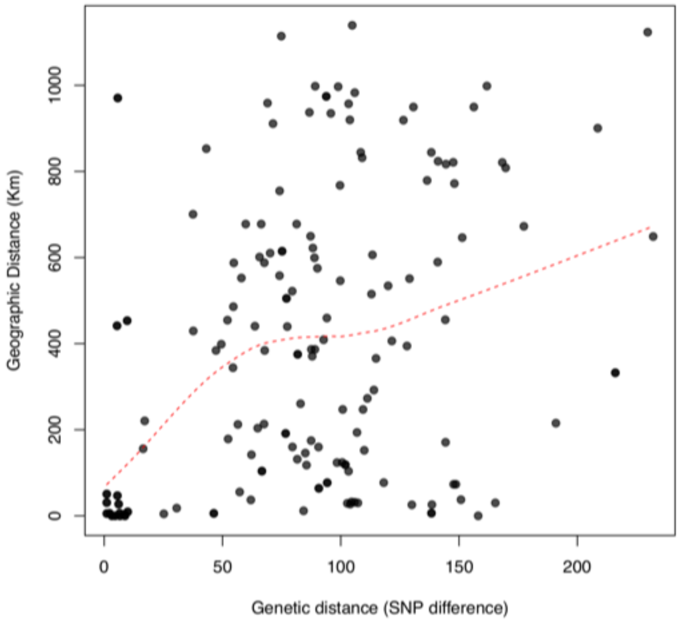

**Supplementary figure 4**

**Phylogenetic tree using Philippine isolates and closely related isolates from previously published datasets*. The posterior probability for the ancestral state reconstruction of country of isolate collection is shown through the pie charts on internal nodes.**

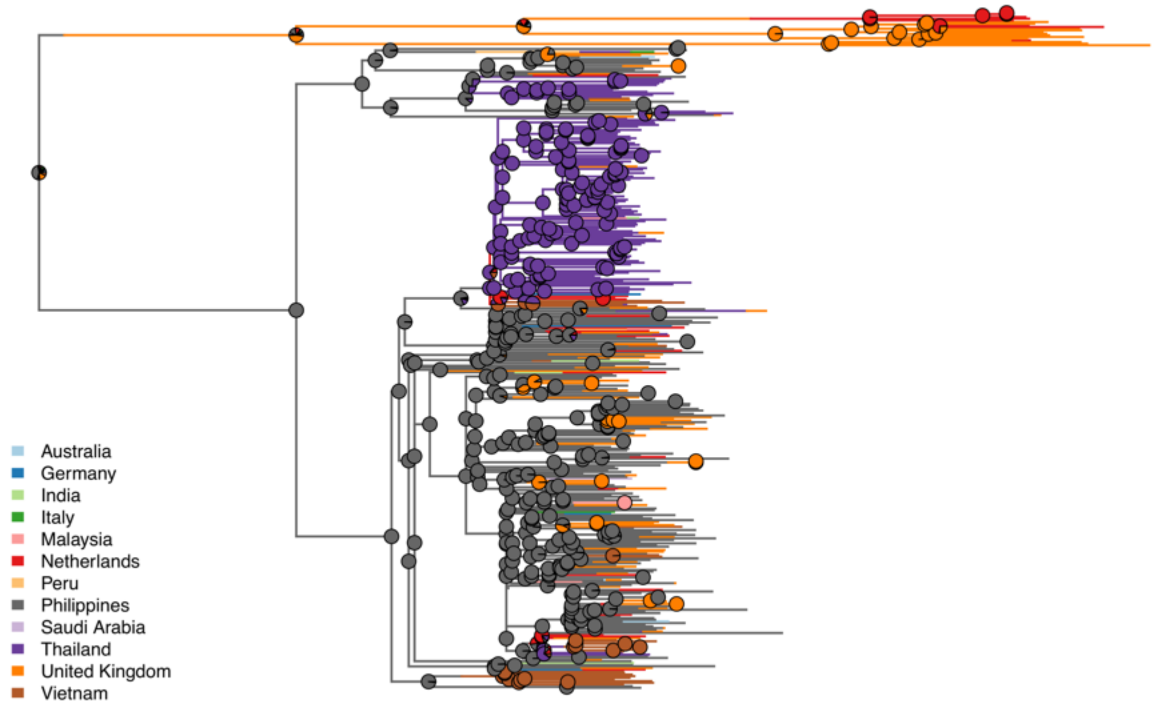


*** see Supplementary data 3**

**Supplementary figure 5**

**Locations of the missense mutations on the *gid* protein (model obtained from Chopin database). The S-adenosyl-L-methionine binding motif is highlighted in blue. The locations of the mutations are highlighted in red.**

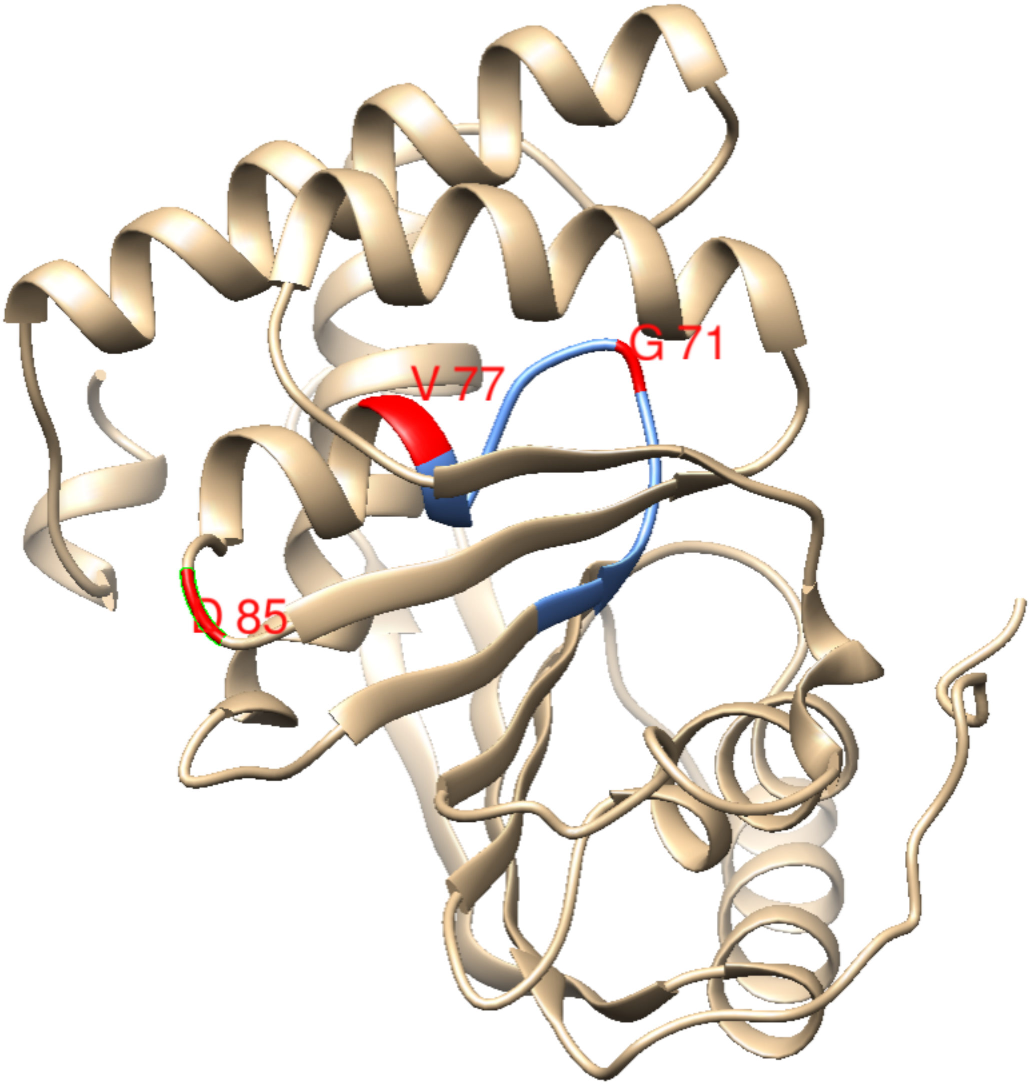

**Supplementary table 1**

**The sensitivity and specificity of drug resistance prediction based on whole genome sequencing**

| Drug | Total | Susceptible | Resistant | Sensitivity | Specificity |
|---|---|---|---|---|---|
| Ethambutol | 155 | 145 | 10 | 90.0% | 95.9% |
| Isoniazid | 139 | 75 | 64 | 95.3% | 98.7% |
| Rifampicin | 154 | 116 | 38 | 97.4% | 100% |
| Streptomycin | 153 | 128 | 25 | 68.0% | 95.3% |
| Amikacin | 178 | 176 | 2 | - | - |
| Capreomycin | 164 | 162 | 2 | - | - |
| Kanamycin | 178 | 170 | 8 | - | - |
| MDR-TB | 135 | 101 | 34 | 94.1% | 100% |
| XDR-TB | 134 | 134 | 0 | - | - |

MDR-TB = multi-drug resistant; XDR-TB = extensively-drug resistant
